# Targeting the insulin growth factor-1 receptor with fluorescent antibodies enables high resolution imaging of human pancreatic cancer in orthotopic mouse models

**DOI:** 10.18632/oncotarget.7576

**Published:** 2016-02-22

**Authors:** Jeong Youp Park, Jin Young Lee, Yong Zhang, Robert M. Hoffman, Michael Bouvet

**Affiliations:** ^1^ Department of Surgery, University of California San Diego, San Diego, CA, USA; ^2^ AntiCancer, Inc., San Diego, CA, USA; ^3^ Department of Internal Medicine, Yonsei University College of Medicine, Seoul, Korea; ^4^ Surgical Service, VA San Diego Healthcare System, San Diego, CA, USA

**Keywords:** insulin-like growth factor-1 receptor (IGF-1R), fluorescent antibody, orthotopic nude mice, imaging, pancreatic cancer

## Abstract

The goal of the present study was to determine whether insulin-like growth factor-1 receptor (IGF-1R) antibodies, conjugated with bright fluorophores, could enable visualization of pancreatic cancer in orthotopic nude mouse models. IGF-1R antibody (clone 24-31) was conjugated with 550 nm or 650 nm fluorophores. Western blotting confirmed the expression of IGF-1R in Panc-1, BxPC3, and MIAPaCa-2 human pancreatic cancer cell lines. Labeling with fluorophore-conjugated IGF-1R antibody demonstrated fluorescent foci on the membrane of the pancreatic cancer cells. Subcutaneous Panc-1, BxPC-3, and MIA PaCa-2 tumors became fluorescent after intravenous administration of fluorescent IGF-1R antibodies. Orthotopically-transplanted BxPC-3 tumors became fluorescent with the conjugated IGF-1R antibodies, and were easily visible with intravital imaging. Gross and microscopic ex vivo imaging of resected pancreatic tumor and normal pancreas confirmed that fluorescence indeed came from the membrane of cancer cells, and it was stronger from the tumor than the normal tissue. The present study demonstrates that fluorophore-conjugated IGF-1R antibodies can visualize pancreatic cancer and it can be used with various imaging devices such as endoscopy and laparoscopy for diagnosis and fluorescence-guided surgery.

## INTRODUCTION

For pancreatic cancer, CA19-9 is the only biomarker being used in the clinic despite many needs for others [1-3]. Several cell surface biomarkers for molecular imaging have been reported in pancreatic cancer [4-6]. We previously demonstrated that CEA, CA19-9, and MUC1 could be targeted in pancreatic cancer with specific fluorescent antibodies, enabling imaging in orthotopic mouse models [7-9].

Type I insulin-like growth factor receptor (IGF-1R) is a potential diagnostic and therapeutic biomarker of several cancers [10]. It is a transmembrane tyrosine kinase receptor comprising two α and two β chains. IGF-1R is the major receptor for IGF-I and IGF-II, and importantly expressed in 97.6% of pancreatic cancers [11]. There is also a report that the expression of IGF-1R in pancreatic cancer is higher than in normal pancreas [12]. IGF-1R plays important roles in cell proliferation, apoptosis, angiogenesis, and tumor invasion [13-15]. In addition, a few studies have shown fluorescence imaging using IGF-1R targeting on tumors is possible, and its potential clinical usefulness has been discussed [16]. In the present study, we demonstrate that fluorescent anti-IGF-1R antibodies target pancreatic cancer in orthotopic mouse models enabling non-invasive imaging and high resolution intra-vital imaging of the tumor.

## RESULTS

### Expression of IGF-1R in pancreatic cancer cells in vitro

**Figure 1:**
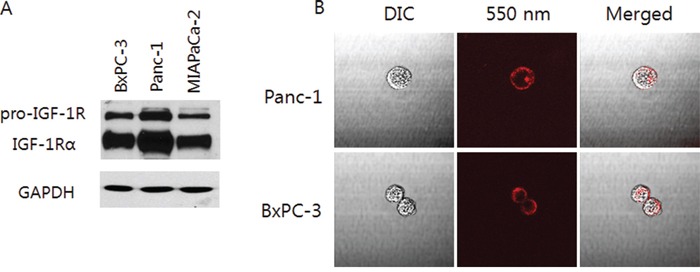
Characterization of pancreatic cancer cell lines **A.** Western blot analysis shows pro-IGF-1R and IGF-1Rα expression at 200 and 130 kDa in pancreatic cancer cell lines, respectively (Panc-1, BxPC-3, MIA PaCa-2). **B.** Labeling of live Panc-1 and BxPC-3 cells with 550 nm fluorophore-conjugated antibodies shows multiple fluorescent foci on the cell membrane. Merged images were created with corresponding DIC (differential interference contrast) images (x60 water immersion objective with the FV1000 confocal microscope, using the 559 nm laser).

Western blotting showed Panc-1, BxPC-3, and MIA PaCa-2 pancreatic cancer cell lines expressed IGF-1R (Figure 1A). After incubation with the fluorophore-conjugated IGF-1R antibody, without permeation, multiple fluorescent foci were visualized on the membrane of Panc-1 and BxPC-3 cells under fluorescence microscopy (Figure 1B) confirming the expression of IGF-1R on cancer cells.

### Targeting subcutaneous pancreatic tumors in nude mice with fluorescent IGF-1R antibodies

**Figure 2:**
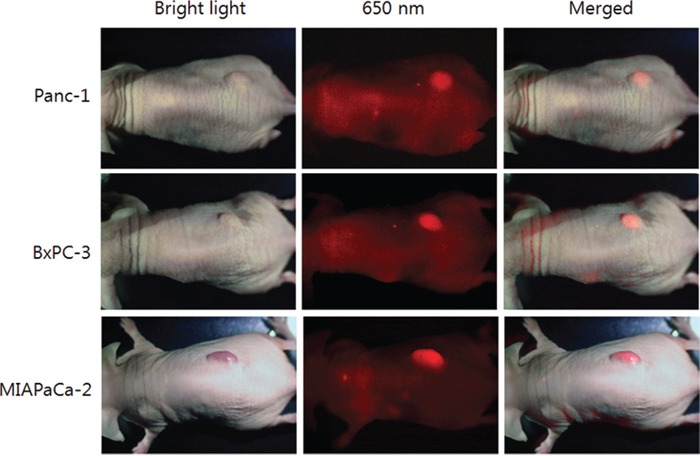
Imaging of 650 nm fluorophore-conjugated IGF-1R antibody targeted subcutaneous pancreatic tumors in vivo The mouse is imaged under both white light and fluorescence illumination with the OV100. The intensity of fluorescence from the Panc-1, BxPC-3, MIA PaCa-2 subcutaneous tumors are stronger than background.

When Panc-1, BxPC-3, and MIA PaCa-2 subcutaneous tumors reached approximately 10 mm in diameter, a single 30 μg dose of DyLight 650-conjugated anti-IGF-1R (clone 24-31) in 150 μl PBS was injected via the tail vein. The mice were imaged by both bright-field and fluorescence illumination using the variable magnification OV100 48 hours after the injection. All the subcutaneous tumors had stronger fluorescence compared to background (Figure 2).

### Targeting orthotopic pancreatic tumors in nude mice with fluorescent IGF-1R antibodies

**Figure 3:**
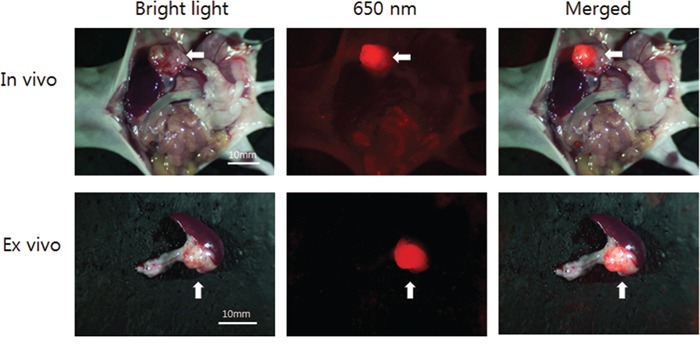
Imaging of fluorophore-conjugated IGF-1R antibody targeted orthotopically-transplanted BxPC3 pancreatic tumors in vivo and ex vivo Fluorescence from orthotopically-transplanted pancreatic tumors in the tail of the pancreas was detected in vivo with the OV100 after abdominal laparotomy. Weak fluorescence was also detected from the skin and, bladder and intestinal contents, but at much lower intensity than the tumor. Also, fluorescence from the resected tumor was detected at high resolution with the IV100, but not from the normal pancreas. White arrows indicate pancreatic tumor.

Mice with BxPC-3 tumors, orthotopically-implanted in the tail of the pancreas, were injected with DyLight 650-conjugated anti-IGF-1R (clone 24-31) antibody. A single 30 μg dose in 150 μl PBS was injected via the tail vein 14 days after tumor implantation. After opening the abdomen, the OV100 detected the bright tumor fluorescence (Figure 3). Minimal fluorescence was also observed at the skin, bladder, and intestinal contents which had lower intensity than the tumor.

### Ex-vivo microscopic imaging of orthotopic pancreatic tumors with fluorescent IGF-1R antibodies

**Figure 4:**
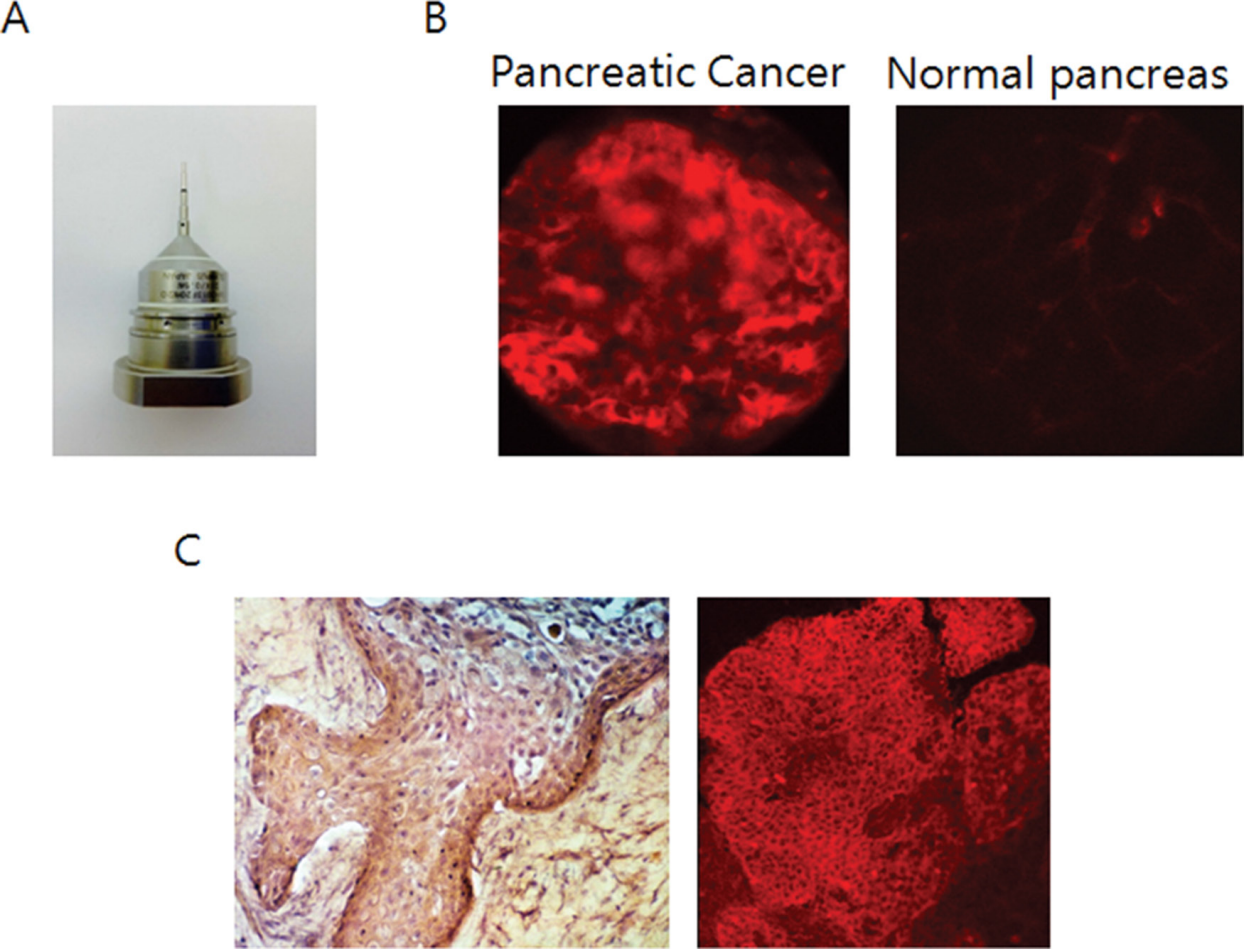
Ex-vivo microscopic imaging of an orthotopic pancreatic tumor labeled with fluorescent anti-IGF-1R antibody **A.** Microprobe lens (x20) used with the IV-100. **B.** Ex vivo imaging done with the IV-100 visualized fluorescence from the surface of BxPC-3 cancer cells in the orthotopic tumor with anti-IGF-1R conjugated to a 650 nm DyLight dye. **C.** H & E and fluorescence staining of a tissue sample (x200) confirm the specific expression of IGF-1R on the surface of the pancreatic cancer cells.

Mice with BxPC-3 tumors, orthotopically-implanted in the tail of the pancreas were injected with a single 10 μg dose of DyLight 650-conjugated anti-IGF-1R (clone 24-31) in 150 μl PBS dose via the tail vein 14 days after tumor implantation. After opening the abdomen, the tumor, normal pancreas and spleen were resected as a block. They were observed under the IV-100 scanning laser microscope with a microprobe objective lens (1.3 mm diameter) (Figure 4A). A strong fluorescence signal on the surface of cancer cells was detected at high resolution with only minimal fluorescence in the interstitium of the normal pancreas (Figure 4B). Hematoxylin & eosin staining of tissue samples and immuno fluorescence staining confirmed the expression of IGF-1R in the pancreatic cancer tissues (Figure 4C).

## DISCUSSION

In the present study, we used a microprobe objective lens for ex vivo imaging, which had x20 magnification power and a diameter of 1.3 mm. It resembles a confocal laser endomicroscope which has a diameter of 0.8 ∼ 2.8mm [17]. Our results suggest microscopic fluorescence imaging using such a device could allow detection of IGF-1R in vivo without tissue sampling.

Recent progress in fluorescence imaging and devices has allowed finer visualization of not only hollow organs such as the esophagus, stomach and colon, but also intra-abdominal solid organs such as the liver, spleen, and pancreas [18-21]. Fluorescence imaging for pancreatic cancer can be especially useful since it can reduce the time and errors associated with tissue biopsy. Since IGF-1R is very frequently expressed in pancreatic cancer, and its expression is stronger than in the normal pancreas [11, 12], detecting IGF-1R can be used for diagnosis purposes. We have previously shown that fluorescence guided surgery (FGS) can increase survival or effect cures in pancreatic cancer mouse models [22]. Targeting IFG-1R in pancreatic cancer with fluorophore conjugated antibodies may also be useful for fluorescence-guided surgery.

IGF-1R fluorescent antibody binding to pancreatic cancer cells and tumors is specific, as shown by Western blotting of IGF-1R binding to the cancer cell membranes (Figure 1) by the much greater antibody-derived fluorescence, compared to non-specific autofluorescence, and in orthotopic as well as subcutaneous tumors seen by both microscopic as well as endoscopic-like imaging devices, and by histological experiments which showed that the antibody specifically binds to tumor tissue (Figure 4).

The present study is a major advance over previous studies [16, 23] on binding of IDG-1R fluorescent antibodies to pancreatic cancer as our model is orthotopic and therefore clinically relevant, including the fact that an endoscope-like device can detect the fluorescent IGF-1R antibody bound to tumor tissue.

With regard to the choice of fluorophore to conjugate to IGF-1R antibodies, we used DyLight 650 and 550 dyes because our previous studies demonstrated that these longer-wavelength dyes had increased depth of penetration and ability to detect the smallest tumor deposits and provided the highest tumor-background ratios (TBRs), resistance to hemoglobin quenching, and specificity compared to shorter wavelength dyes [24]. Other fluorophores may be useful in terms of colors and chemistry as outlined by Kobayashi et al [25, 26].

The tumor-targeting technology described in the present report can be used along with previously developed tumor-targeting strategies [27-32].

## MATERIALS AND METHODS

### Pancreatic cancer cell lines

The human pancreatic cancer cell lines BxPC-3 [33] and Panc-1 [34] MIA PaCa-2 were maintained in RPMI 1640 medium and DMEM supplemented with 10% fetal bovine serum (Hyclone, Logan, UT), and penicillin/streptomycin (Gibco-BRL, Carlsbad, CA). All cells were cultured at 37° C in a 5% CO_2_ incubator.

### Mice

Athymic *nu/nu* nude mice (AntiCancer Inc., San Diego, CA), 4–6 weeks old, were used in the study. Mice were kept in a barrier facility under HEPA filtration and fed with an autoclaved laboratory rodent diet. All mouse surgical procedures and imaging were performed after anesthetized by intramuscular injection of 50% ketamine, 38% xylazine, and 12% acepromazine maleate (0.02 ml). Animals received buprenorphine (0.10 mg/kg ip) immediately prior to surgery and once a day over the next 3 days. The maximum tumor size allowed to grow was 2 cm. The condition of the animals was monitored every day. CO2 inhalation was used for euthanasia. To ensure death following CO_2_ asphyxiation, cervical dislocation was performed. All animal studies were approved by AntiCancer, Inc.'s Institutional Animal Care and Use Committee (IACUC) in accordance with the principals and procedures outlined in the National Institute of Health Guide for the Care and Use of Animals under Assurance Number A3873-1.

### Antibody-dye conjugation

Mouse monoclonal antibodies to IGF-1R (clone 24-31; Thermo Scientific, Rockford, IL, USA) were conjugated with DyLight 650 or 550 dyes (Thermo Scientific, Rockford, IL, USA) per manufacturer specifications, ensuring a minimum of at least 4:1 dye: protein ratio. Protein: dye concentrations were confirmed using a NanoDrop Spectrophotometer (Thermo Fisher Scientific, Waltham, Massachusetts) [24].

### Western blotting

Cell lysates were extracted in lysis buffer containing 70 mM β-glycerophosphate, 0.6 mM sodium orthovanadate, 2 mM MgCl_2_, 1 mM ethylene glycol tetraacetic acid, 1 mM DTT (Invitrogen, Grand Island, NY, USA), 0.5% Triton-X100, 0.2 mM phenylmethylsulfonyl fluoride, and 1% protease inhibitor cocktail (Sigma-Aldrich, St. Louis, MO, USA). Lysates were separated by sodium dodecylsulfate–polyacrylamide gel electrophoresis (SDS-PAGE) and transferred to polyvinylidene fluoride membranes (Millipore, Billerica, MA, USA). The membranes were blocked in 5% (w/v) non-fat dry milk and probed with anti-IGF-1R (SC-712; Santa Cruz, Dallas, TX, USA). The immunoreactive proteins were visualized using the SuperSignal West Pico Chemiluminescent Substrate (Thermo Scientific).

### Labeling of live cells in vitro using fluorescent IGF-1R antibodies

Panc-1 and BxPC-3 cells (2 × 10^5^) were cultured overnight. Anti-IGF-1R (clone 24-31) conjugated with DyLight 550 dye was diluted to 4 μg/ml in phosphate-buffered saline (PBS, Corning Cellgro, Manassas, VA). The culture medium from the cells was aspirated and the diluted antibody was added to the live cells. Cells were incubated with antibody for 1 hour at room temperature. The cells were washed gently 2 times with PBS after the antibody was aspirated. The cells were observed under an FV1000 confocal microscope (Olympus, Tokyo, Japan) with white light and 559 nm laser [35].

### Immunohistochemistry

Anti-IGF-1R (clone 24-31) conjugated with DyLight 650 was used for staining tumor sections on slides. The slides were incubated with 10% normal donkey serum for 1 hour at room temperature, and incubated with the conjugated antibody at room temperature for 1 hour at a dilution of 1:100. Tissues were dried and observed with an IV-100 scanning laser microscope (Olympus, Tokyo, Japan) with a 633 nm laser [36]. Alternate slides from the same frozen tumor tissue were stained with hematoxylin and eosin and observed under light microscopy.

### Subcutaneous and orthotopic tumor mouse models

To make subcutaneously-transplanted pancreatic tumor models, Panc-1, BxPC-3, and MIA PaCa-2 human pancreatic cancer cells (2 × 10^6^) were injected subcutaneously into the flanks of nude mice. When the subcutaneous tumors grew between 10 and 20 mm in diameter, imaging of the subcutaneous tumor was performed. To make orthotopic-tumor mouse models, subcutaneous tumors were harvested and divided into small fragments which were implanted onto the tail of the pancreas using 8-0 nylon sutures in nude mice, as previously described [37-40].

### Whole body and intra-vital imaging

In both subcutaneous and orthotopic tumor models, the mice were injected with the fluorescent antibody in the tail vein. After 48 hours, whole body non-invasive and intra-vital imaging of the subcutaneous and orthotopic tumors was performed using the OV100 Small Animal Imaging System (Olympus, Tokyo, Japan) [41]. The optimal dose for animal studies was decided by the amount of the conjugated antibody which produced images with the best TBR in the subcutaneous-tumor model.

### Intravital laser scanning microscope

The IV-100 (Olympus) and the conjugated antibody were used to obtain microscopic fluorescence images of pancreatic tumors and the normal pancreas in the orthotopic mouse model. The IV-100 operates with four lasers (488, 561, 633, and 748 nm) for excitation; three of which can be used simultaneously for imaging. Its microprobe lens has an external diameter of 1.3 mm and delivers high resolution images in the visible and near-infrared spectrum. Due to its small size, it can be used to image abdominal organs through a small incision [42].

### Image analysis

All images were analyzed using Image-J (National Institutes of Health, Bethesda, Maryland) before the process of images and compared. Image process was done with Adobe Photoshop CS3 (Adobe Systems Inc., San Jose, California).
